# Solid lipid nanoparticles to improve bioaccessibility and permeability of orally administered maslinic acid

**DOI:** 10.1080/10717544.2022.2086937

**Published:** 2022-06-28

**Authors:** Aixa Aguilera-Garrido, Elena Arranz, María José Gálvez-Ruiz, Juan Antonio Marchal, Francisco Galisteo-González, Linda Giblin

**Affiliations:** a Department of Applied Physics, University of Granada, Granada, Spain; b Excellence Research Unit “Modeling Nature” (MNat), University of Granada, Granada, Spain; c Teagasc Food Research Centre, Moorepark, Fermoy, Ireland; d Biopathology and Regenerative Medicine Institute (IBIMER), Centre for Biomedical Research (CIBM), University of Granada, Granada, Spain; e Instituto de Investigación Biosanitaria ibs.GRANADA, University Hospitals of Granada – University of Granada, Granada, Spain; f BioFab i3D – Biofabrication and 3D (Bio)Printing Laboratory, University of Granada, Granada, Spain; g Department of Human Anatomy and Embryology, Faculty of Medicine, University of Granada, Granada, Spain

**Keywords:** Solid lipid nanoparticle, maslinic acid, digestion, bioaccessibility, intestinal permeability

## Abstract

Maslinic acid (MA) is a plant-derived, low water-soluble compound with antitumor activity. We have formulated MA in the form of solid lipid nanoparticles (SLNs) with three different shell compositions: Poloxamer 407 (PMA), dicarboxylic acid-Poloxamer 407 (PCMA), and HA-coated PCMA (PCMA-HA). These SLNs improved the solubility of MA up to 7.5 mg/mL, are stable in a wide range of pH, and increase the bioaccessibility of MA after *in vitro* gastrointestinal (GI) digestion. Gastrointestinal digested SLNs afforded MA delivery across *in vitro* gut barrier models (21 days old Caco-2 and mucus-producing Caco-2/HT29-MTX co-cultures). The cellular fraction of Caco-2/HT29-MTX co-cultures retained more MA from GI digested PCMA-HA than the Caco-2 monolayers. The concentration of MA reached in the basolateral chamber inhibited growth of pancreatic cancer cells, BxPC3. Finally, confocal microscopy images provided evidence that Nile Red incorporated in MA SLNs was capable of crossing Caco-2 monolayers to be taken up by basolaterally located BxPC3 cells. We have demonstrated that SLNs can be used as nanocarriers of hydrophobic antitumor compounds and that these SLNs are suitable for oral consumption and delivery of the bioactive across the gut barrier.

## 1. Introduction

Maslinic acid (MA) is a pentacyclic triterpene that exhibits antitumor, antioxidant, anti-inflammatory, antidiabetic, antiparasitic, cardioprotective and neuroprotective properties (Lozano-Mena et al., 2014). The interest in MA as a nutraceutical in the cancer research area arises from its protective role in the initial stages of carcinogenesis in colorectal cancer (Sánchez-Tena et al., 2013; Juan et al., 2019; Wei et al., 2019). MA inhibits proliferation and promotes apoptosis in several cancer cells types, including lung cancer cells (Bai et al., 2016), human colorectal cancer cells (Juan et al., 2008; Reyes-Zurita et al., 2009; Wei et al., 2019), bladder cancer cells (Zhang et al., 2014), renal cell carcinoma (Thakor et al., 2017) and pancreatic cancer cells (Zhang et al., 2021). It has been reported to inhibit angiogenesis (Thakor et al., 2017) and cell migration and invasion in several cancers (Wei et al., 2019; Zhang et al., 2021). Moreover, MA can modulate the inflammatory response and potentiate the immune system action against cancer cells (Sánchez-Quesada et al., 2015; Lai et al., 2019). Sánchez-Quesada et al. reported that MA promoted the *in vitro* production of pro-inflammatory and macrophage recruitment-related cytokines in macrophages and that it favored their differentiation to M1 state, which is the phenotype involved in the immune defense against cancer cells (Sánchez-Quesada et al., 2015). Finally, the MA-supplemented diet prevented denervation-induced loss of skeletal muscle mass and strength, i.e. prevented skeletal muscle atrophy, in a sciatic-nerve transection mice model through the downregulation of the TGF-β signaling pathway, whose overexpression is linked to cancer cachexia-induced muscle atrophy (Yamauchi et al., 2021). Therefore, administration of MA in the diet to cancer patients could reduce muscle atrophy induced by cachexia. However, this natural lipid present in several plant sources is a low water-soluble compound (3.6 µg/L) (Medina-O’Donnell et al., 2017). This low solubility feature limits its oral bioaccessibility and bioavailability. Nonetheless, the oral administration route is the preferred and more extended drug administration route, because it supports high patient compliance.

Nanoparticles have routinely been employed to increase the solubility of poorly water-soluble substances (Thanki et al., 2013; Williams et al., 2013; Wang et al., 2014; Hsu et al., 2019). In the case of orally administered nanoparticles, the improvement in solubility for the hydrophobic compound has a knock on improvement on its bioaccessibility (Silva et al., 2018; Ban et al., 2020; Aguilera-Garrido et al., 2021) and bioavailability across the gut barrier (Andey et al., 2015; Fofaria et al., 2016; Gao et al., 2016; Sun et al., 2016; Ban et al., 2020). Solid lipid nanoparticles (SLNs) are lipid-cored nanocarriers that are solid at body temperature. The main advantage of SLNs, over and above oily cored lipid systems, is their sustained drug release over time avoiding a sudden release of the encapsulated compound (Zur Mühlen et al., 1998; Mehnert & Mader, 2001). The special characteristics of the gastrointestinal (GI) tract (i.e. acidic gastric pH, pH change, and enzymatic digestion) make oral administration of nanoparticles as drug delivery systems, a challenge. Some solutions include the use of polymeric coatings of SLNs to reduce the incidence of gastric burst release (Ganesan et al., 2018). The shell composition influences the colloidal stability of the system, as well as the interaction with enzymes, bile salts, and the mucus protective layer of the intestinal cells (Thanki et al., 2013).

In view of the potential beneficial biological activities of MA, we propose the formulation of MA in the form of SLNs to improve the solubility and bioavailability of MA, but also as a potential nanocarrier of hydrophobic drugs. We have synthesized and characterized three SLNs with a MA core but differing in their coatings: Poloxamer 407 (P407), dicarboxylic-P407, and hyaluronic acid (HA). Poloxamer 407, also known as Pluronic^®^ F127, is a nonionic triblock copolymer. It is a generally recognized as safe (GRAS) excipient composed of two hydrophilic poly-ethylene oxides (PEO) blocks, which flank a central hydrophobic poly-propylene oxide (PPO) block. This chemical structure provides the molecule with excellent surface-active properties. Dicarboxylic acid P407 has two terminal carboxyl groups that afford an extra negative charge at the surface and allow the possibility of covalent binding to different chemical groups, the most commonly used being the linking to amino groups through the well-established carbodiimide method (Punfa et al., 2012). Nanoparticles stabilized with poloxamers have shown a reduction in the adsorption of serum proteins known as protein-corona. Thus, P407 can help to modulate the formation of this protein-corona, allowing nanoparticles to have longer blood circulation times (Sánchez-Moreno et al., 2015). P407 coating also improves the mucus penetration of nanoparticles, i.e. the diffusion of the nanoparticles through the intestinal mucus layer (Schattling et al., 2017). Designing nanocarriers capable of penetrating the intestinal mucus layer will presumably favor their permeability across the gut barrier. Alternatively, nanocarriers with mucoadhesion properties facilitate its retention in the mucus layer allowing sustained drug delivery (Cuggino et al., 2019). HA is a biocompatible, biodegradable, and nontoxic glycosaminoglycan, composed of *N*-acetyl-d-glucosamine and d-glucuronic acid, which has mucoadhesive properties and may assist SLNs bioavailability across the gut barrier (Lim et al., 2000; Fallacara et al., 2018). Moreover, HA also specifically binds to CD44 and RHAMM receptors, both of which are overexpressed by cancer stem cells (Fallacara et al., 2018).

Permeability across the gut barrier can be evaluated in the laboratory by the use of intestinal epithelium cell monolayers capable of forming tight junctions. The human colonic epithelial cell line Caco-2 is routinely used as a gut barrier model as it matures over 21 days to form polarized monolayers with distinct apical and basolateral faces (Verhoeckx et al., 2015). *In vivo*, the gut barrier is composed of several cell types including goblet cells responsible for mucus production. The co-culturing of Caco-2 cells with the human colon cancer cell line HT29 treated with methotrexate (HT29-MTX) introduces polarized goblet cells to the Caco-2 monolayer (Verhoeckx et al., 2015).

The present study compares the behavior of three MA SLNs with different interfacial characteristics. Their stability under *in vitro* simulated GI conditions was analyzed. The interaction of SLNs with two different intestinal *in vitro* models (Caco-2 model and a mucus-producing Caco-2/HT29-MTX model) was then assessed to investigate permeability across the gut barrier to impact target cancer cells.

## 2. Materials and methods

### 2.1. Materials

MA (crategolic acid or 2α,3β-dihydroxyolean-12-en-28-oic, C_30_H_48_O_4_, molecular weight 472.7 g/mol) was extracted from olive oil production by-products and purified as previously described (purity >80%) (García-Granados Lopez de Hierro, 1997). Briefly, MA was successively extracted in a Soxhlet with hexane and ethyl acetate, obtaining a solid residue. MA was then purified from the solid residue by column chromatography over silica gel, eluting with a dichloromethane/acetone gradient of increasing polarity, starting with a 40:1 ratio.

Poloxamer 407 (Pluronic F127, [CAS:9003-11-6]), *N*-hydroxysulfosuccinimide sodium salt (sNHS, REF 56485, (CAS [106627-54-7]), hexamethylenediamine (CAS [124-09-4]), pepsin (P6887), 4-morpholineethanesulfonic acid (MES hydrate, M8250), Nile Red (72485), porcine pancreatin (P7545), bovine bile (B3883), Hank's Balanced Salt Solution (HBSS, H8264), DAPI (D9542), and thiazolyl blue tetrazolium bromide (MTT, M2128, CAS [298-93-1]) were purchased from Sigma-Aldrich, Merck (Darmstadt, Germany). Rabbit gastric extract (RGE25) was supplied by Lipolytech (Marseille, France). HA (1900 kDa) was supplied by BioIbérica (Barcelona, Spain). Analytical grade ethanol, acetone, and propanol were commercially supplied by Sigma-Aldrich, Merck (Darmstadt, Germany). Ethyl acetate (AC01481000, CAS [141-78-6]) was purchased from Scharlab (Barcelona, Spain). 1-(3-Dimethylaminopropyl)-3-ethylcarbodiimide hydrochloride (EDCI, A10807, CAS [25952-53-8]) was purchased from Alfa Aesar (Thermo Fisher, Dreieich, Germany). Dicarboxylic acid Poloxamer 407 was synthesized and kindly donated, by Dr. A. Parra at the Organic Chemistry Dept. of the University of Granada, as previously described (Punfa et al., 2012). Biotech CE Dialysis Tubing 300 kDa (31MM, 131456) was purchased from Repligen (Ravensburg, Germany). CellTiter 96 Aqueous One Solution reagent (3-(4,5-dimethylthiazol-2-yl)-5-(3-carboxymethoxyphenyl)-2-(4-sulfophenyl)-2H-tetrazolium, MTS, G358C) was purchased from Promega (MyBio, Kilkenny, Ireland). Tissue culture coverslips (adherent, sterile, 13 mm (83.1840.002)) were purchased from Sarstedt (Newton, NC). Phosphate-buffered saline (PBS) pH 7.4 (Medicago, 09-8912-100) was provided by Labortecnic (Granada, Spain).

The enzymatic activity and the bile salt concentration were determined according to the INFOGEST protocol specifications (Brodkorb et al., 2019). All other reagents unless specified were purchased from Sigma (Wicklow, Ireland).

### 2.2. Cell lines and culture conditions

Caco-2 cells (American Tissue Culture Collection HTB-37™) were cultured in Dulbecco’s modified Eagle’s medium high glucose content (DMEM-high glucose) supplemented with 10% fetal bovine serum (FBS) and antibiotics (100 U/mL penicillin and 100 μg/mL streptomycin). HT29-MTX (derived from HT29 sourced from American Tissue Culture Collection HTB-38™) were cultured in the same medium with 1% non-essential amino acids. BxPC3 (American Tissue Culture Collection CRL-1687™) was cultured in Roswell Park Memorial Institute 1640 Medium (RPMI-1640) supplemented with 10% FBS, antibiotics (100 U/mL penicillin and 100 μg/mL streptomycin) and l-glutamine. Cell passage numbers for Caco-2 ranged from 20 to 40, for HT29-MTX from 60 to 75 and for BxPC3 from 10 to 20.

### 2.3. Synthesis and colloidal characterization of SLNs

MA SLNs were synthesized by using a solvent displacement method. Briefly, 60 mL of an organic phase composed of a 1:1 mixture of ethanol:acetone containing MA (2.5 mg/mL) were mixed with an equal volume of aqueous phase containing the surfactant (P407 or dicarboxylic P407, 1 mg/mL), to form P407 + MA (PMA) or dicarboxylic acid P407 + MA (PCMA) nanoparticles. The solutions became turbid immediately after the mixing of aqueous and organic phases, owing to the SLNs formation. Then, solvents were removed in a rotary evaporator at 40 °C (until a final volume of 20 mL) and excess poloxamer was removed by dialyzing against distilled water using 300 kDa molecular weight cutoff cellulose dialysis tubing for 48 h at 4 °C.

Similarly, Nile Red loaded-MA SLNs were prepared but with the addition of 0.0025 mg/mL of Nile Red (0.1% w/w respect to MA) in the organic phase.

PCMA nanoparticles were further functionalized with HA (PCMA-HA). To achieve the coating, we performed two consecutive covalent bindings by means of the carbodiimide reaction. First, carboxyl groups from HA were bound to amino groups from 1,6-hexanediamine. Then, this HA with amino groups was covalently linked to the carboxyl groups from the PCMA. For the covalent linking between HA and 1,6-hexanediamine, HA was activated by mixing 3.95 × 10^−2^ mmol COOH from HA (3 mL at 5 mg/mL, in MES buffer 0.1 M, pH 4) with 7.7 × 10^−2^ mmol EDCI (at 49.8 mg/mL, in MES buffer 0.1 M, pH 4) and 7.7 × 10^−2^ mmol of sNHS (at 24 mg/mL, in MES buffer 0.1 M, pH 4). The reaction mix was kept at room temperature under magnetic stirring for 1 h. Then, 77 × 10^−2^ mmol of 1,6-hexanediamine (at 60 mg/mL, in water) were added to the reaction. The pH was adjusted at 8.7, and the reaction was kept under magnetic stirring at room temperature for 2 h. The reaction product, HA-hexanediamine, was dialyzed against water (300 kDa molecular weight cutoff, 48 h at 4 °C) and lyophilized.

For the covalent linking between PCMA and HA-hexanediamine, a volume of PCMA containing 1.2 × 10^−3^ mmol of dicarboxylic acid P407 was activated by adding 4.8 × 10^−3^ mmol of EDCI (at 49.8 mg/mL, in MES buffer 0.1 M, pH 4) and 4.8 × 10^−3^ mmol of sNHS (at 24 mg/mL, in MES buffer 0.1 M, pH 4). After 1 h at room temperature and under magnetic stirring, pH was raised to 8 and HA-hexanediamine (the quantity equivalent to 3.95 × 10^−2^ mmol of COOH from HA) was added to the PCMA solution. The reaction was kept at room temperature overnight and then centrifuged (21,000×*g*, 30 min) to remove reactant excess and undesired reaction subproducts, and resuspended with distilled water.

The hydrodynamic diameter (*D*_H_), polydispersity index (PDI) and the *ζ*-potential of the SLNs (PMA, PCMA, and PCMA-HA) were determined by dynamic light scattering (DLS) using a Zetasizer Nano-S system (Malvern Instruments, Malvern, UK). Samples were diluted (1:100 ratio) in a pH 7 buffer (KH_2_PO_4_, 1.13 mM). All measurements were performed in triplicate at 25 °C and the self-optimization routine from the instrument software was used. The *ζ*-potential was calculated according to the Smoluchowski theory. Data appears as the mean value ± standard deviation.

SLNs imaging and sample preparation were performed at the Center for Scientific Instrumentation (CIC), University of Granada. SLNs were imaged with high-resolution transmission electron microscopy (HRTEM) Thermo Fisher Scientific TALOS F200X at 120 kV. A volume of 25 µL of each sample was incubated on carbon-coated grids for five minutes before being washed off with ultra-pure water. Uranyl acetate was employed for negative stain samples. Three images were taken per sample.

### 2.4. Quantification of MA and Nile red from the SLNs

SLNs were diluted in a 1:100 ratio with distilled water. A volume of 10 µL of diluted nanoparticles was added to 1 mL of 2-propanol, vortexed for 1 min and incubated on an ultrasound water bath for 10 min. Then, samples were centrifuged for 10 min at 21,000×*g*, the supernatant was collected and analyzed by liquid chromatography-electrospray ionization-tandem mass spectrometry (LC-ESI-MS/MS). Analytes were separated using an ACQUITY UPLC^®^ BEH C18 (Waters™, Milford, MA) (1.7 µm, 50 × 2.1 mm) column equilibrated with 0.1% formic acid in H_2_O and acetonitrile with 0.1% formic acid (50:50, v/v) at 40 °C. Samples were kept at 20 °C and 5 μL of the sample were injected in the column. Analytes were eluted with acetonitrile with 0.1% formic acid gradient at a flow rate of 0.4 mL/min. The MS analysis was performed in a Waters XEVO-TQS. Ion transitions were monitored with multiple reaction monitoring (MRM) in negative ionization mode for MA (*m/z* 471.58 > 393.34, and *m/z* 471.58 > 405.44) and in positive ionization mode for Nile Red (*m/z* 319.17 > 190.25, and *m/z* 319.17 > 219.01). MA (from 0.093 to 1.5 mg/L) and Nile Red (from 0.001 to 0.015 mg/L) were used as standards.

### 2.5. Static *in vitro* digestion

The *in vitro* GI digestion of SLNs and free MA was performed according to the INFOGEST static *in vitro* digestion protocol which models adult GI digestion (Brodkorb et al., 2019). This is a widely accepted consensus protocol for digestion in the upper adult gut that is widely documented to accurately and truly mimic the *in vivo* situation. A 2 min oral phase, without enzymes, was carried out by adding 5 mg MA or the equivalent volume of SLNs to 2 mL of simulated salivary fluid. The pH was adjusted to 7 with HCl, CaCl_2_ was added to a final concentration of 1.5 mM, and the volume was raised to 5 mL by adding MilliQ H_2_O. For the gastric phase, the 5 mL sample from the oral phase was mixed with 4 mL of simulated gastric fluid. Porcine pepsin (EC 3.4.23.1) and rabbit gastric extract were added to achieve a pepsin activity of 2,000 U/mL and gastric lipase activity of 60 U/mL. Then, pH was adjusted to 3 with HCl, the volume brought to 10 mL, and the samples incubated for 2 h at 37 °C, under continuous shaking. Enzymatic activity was stopped by raising the pH to 7 with NaOH (0.5 M). This gastric sample (10 mL) was mixed with 4 mL of simulated intestinal fluid. Pancreatin (EC 232.468.9) and bile salts were added to achieve a final activity of 200 U/mL and a final concentration of 10 mM, respectively. The final volume reaction was completed with water to 20 mL, and the pH was adjusted to 7. The intestinal sample was incubated for 2 h at 37 °C under continuous shaking, and the reaction was stopped by adding Pefabloc (5 mM final concentration) and Orlistat (1 mM final concentration). Every digestion assay was carried out in triplicate.

Enzymes, bile salts, and enzyme inhibitors were removed by filtration using Amicon^®^ Ultra-15 Centrifugal Filters 100 kDa MWCO (Merck, Darmstadt, Germany, UFC9100). The retentate containing the SNLs was further washed by another process of filtration after adding 10 mL and 20 mL of HBSS to the gastric sample and the intestinal sample, respectively. The concentration of MA in the digested samples after the cleaning process was quantified as specified in Section 2.4.

### 2.6. Toxicity assays

Caco-2 cells were seeded on 96-well plates (5,000 cells/well) and allowed to grow overnight. Test samples were prepared by diluting GI SLNs in DMEM at different MA concentrations (150–400 μM). Cells were then incubated with 100 μL of test sample for 2 h at 37 °C. MTS (20 μL) was added to each well, and the plate was incubated for a further 2 h. After incubation, absorbance was recorded at 490 nm (Synergy HT BioTek, Winooski, VT). Each concentration was assayed in three different wells and the toxicity assays were repeated on three independent days. Positive control was DMEM with no cells and negative control was cells with DMEM. Cellular viability was calculated as a percentage of the negative control.

BxPC3 pancreatic cancer cells were initially seeded on 96-well plates (4,500 cells/well) and allowed to grow overnight. They were then incubated with different concentrations of MA (from 0.9 to 79.7 μM, prepared from a stock solution of MA 1.5 mM and 1% DMSO in RPMI media). Negative and positive controls were RMPI medium with or without cells, respectively. To simulate a continued dosage regimen, the test sample medium was removed and replaced with the corresponding fresh MA-RPMI medium every 12 or 24 h. After 72 h of treatment, test samples were removed from the plates, wells were washed twice with PBS, and 100 μL/well MTT was added. After 3 h of incubation, MTT was removed from the plates, 100 μL/well of DMSO were added and absorbance was recorded at 570 nm using a HEALES MB-580 microplate reader (HEALES, Shenzhen, China). Each test sample was assayed in three different wells and each experiment was replicated on three different days. The data are the mean values ± standard deviation.

### 2.7. *In vitro* intestinal epithelium permeability models

Caco-2 and HT29-MTX cells were grown in Corning^®^ Transwell^®^ cell culture inserts (12 mm diameter with 0.4 μm pore polyester membrane inserts) assembled in Corning^®^ 12-well plates (Corning, Corning, ‎NY). For the Caco-2 intestinal epithelium *in vitro* model, 6 × 10^4^ cells were seeded per insert, whereas for the co-culture model 4.5 × 10^4^ Caco-2 cells (75%) and 1.5 × 10^4^ HT29-MTX cells (25%) were seeded in each insert. Before the seeding, 1.5 mL and 0.4 mL of DMEM-high glucose supplemented with 10% FBS, 5% antibiotics and 5% glutamine, were added on the basolateral and apical chambers, respectively. Plates were incubated at 37 °C for 15 minutes. Then, the cells were added to the apical chamber in a volume of 0.1 mL/insert (final apical volume 0.5 mL). Cells were allowed to grow and differentiate for 21 days with media changes every two days. Transepithelial electrical resistance (TEER) was measured at 37 °C weekly to monitor the evolution of the epithelium monolayer.

For the permeability assay, on day 21 TEER values were recorded, and monolayers with values <550 Ω·cm^2^ were discarded. The culture medium was then removed and cells washed twice with HBSS. Inserts were then transferred to new 12-well plates. A volume of 1.5 mL and 0.25 mL of DMEM-high glucose without phenol red were added to the basolateral and apical chambers, respectively, and plates were incubated again at 37 °C for 30 min. TEER values were checked and then 0.25 mL of the digested SLNs samples in DMEM were added to the apical chamber to give a final apical volume of 0.5 mL. Plates were incubated at 37 °C for 4 h. TEER values were again recorded, and the culture medium from the basolateral and apical chambers was collected for later analysis. Cells were washed twice with PBS, detached from the insert membrane mechanically and collected in 0.5 mL of PBS. Samples were immediately stored at −80 °C. Each test sample was tested in at least four different wells, from at least two independent assays.

### 2.8. Extraction and LC–MS/MS quantification of the MA from the permeability assay samples

The extraction of MA from apical, cellular and basolateral permeability samples was performed according to a previously validated protocol (Peragón et al., 2015). The cellular fraction was washed with PBS and then disrupted by adding 0.5 mL of lysis buffer (20 mM Tris–HCl pH 7.5, 1 mM dithiothreitol (DTT), 1 mM ethylenediaminetetraacetic acid (EDTA), 2% Triton X100, 0.2 mM phenyl methylsulfonyl fluoride (PMSF) and 2% sodium deoxycholate). Cells were lysed using an ultrasonic water bath (Clifton, Thermo Fisher, Dublin, Ireland) for 10 min. Ethyl acetate (1 mL to cell lysate sample, 1 mL to apical samples, and 2 mL to basolateral samples) was then added and samples were vigorously vortexed for 1 min and centrifuged for 5 min at 6500×*g* at 20 °C. The ethyl acetate organic supernatant from each sample was collected in 15 mL vials and the remaining aqueous fraction was extracted five more times (for cell lysates and apical samples) and seven more times (for basolateral samples). The pool of the organic supernatant from each sample was evaporated in a rotary evaporator (Barloworld Scientific Limited, London, UK) under vacuum at 50 °C until dry. The remaining residue was dissolved in 2-propanol (250 μL for basolateral samples, 500 μL for cell lysates, and 750 μL apical samples) and the MA concentration was analyzed by LC–MS/MS as described in Section 2.4.

### 2.9. Confocal fluorescence microscopy

Circular cover glasses for confocal fluorescence microscopy were placed at the bottom of each well in a 12-well plate. Then, 2.5 × 10^4^ BxPC3 cells were seeded into each well and allowed to grow in RPMI for 24 h. Caco-2 monolayer inserts, prepared as stated in Section 2.7, were washed twice with PBS, and transferred to wells with BxPC3 cells pre washed with PBS. DMEM-high glucose without Phenol Red was added to the basolateral (1.5 mL) and apical (0.25 mL) compartments. GI digested Nile Red-loaded MA SLNs (PMA-NR GI, PCMA-NR GI, and PCMA-HA-NR GI) were added to the apical side of the chamber to reach a final apical volume of 0.5 mL and 0.22 nM Nile Red concentration. After 4 h of incubation, the apical and basolateral chambers were washed twice with PBS. Then, transwell Caco-2 membranes and cover glasses with BxPC3 from the basolateral compartment were collected, cells were fixed with PFA (4%) for 20 min and cell nuclei were stained with DAPI (1 μg/mL). As a control, BxPC3 cells were incubated directly with 0.5 mL of GI digested Nile Red-loaded MA SLNs (0.22 nM Nile Red) plus 1.5 mL DMEM for 4 h. These control cells were then washed twice with PBS, fixed with PFA (4%) for 20 min and cell nuclei stained with DAPI (1 μg/mL). Samples were prepared for confocal fluorescence microscopy imaging with Fluoroshield™ histology mounting medium in microscope slides. Specimens were imaged with a Zeiss LSM 710 inverted laser scanning confocal microscope (Zeiss, Oberkochen, Germany) and processed with Zen Lite 3.4 software. Excitation and emission wavelengths were 543 nm and 651, and 405 nm and 450 nm, for Nile Red and DAPI respectively.

### 2.10. Statistical analysis

Data from permeability results were analyzed with a one-way ANOVA test (*p*<.05) followed by Bonferroni’s multiple comparison post hoc test. The statistical analysis was performed with Origin8. The Student statistic *t*-test *p*<0.05, two tail and two sample *t*-test for equal variances were applied to analyze the toxicity of MA on BxPC3 cell line. Data are expressed as the mean value ± standard deviation.

## 3. Results

### 3.1. Synthesis and colloidal characterization of MA SLNs

**Figure 1.**
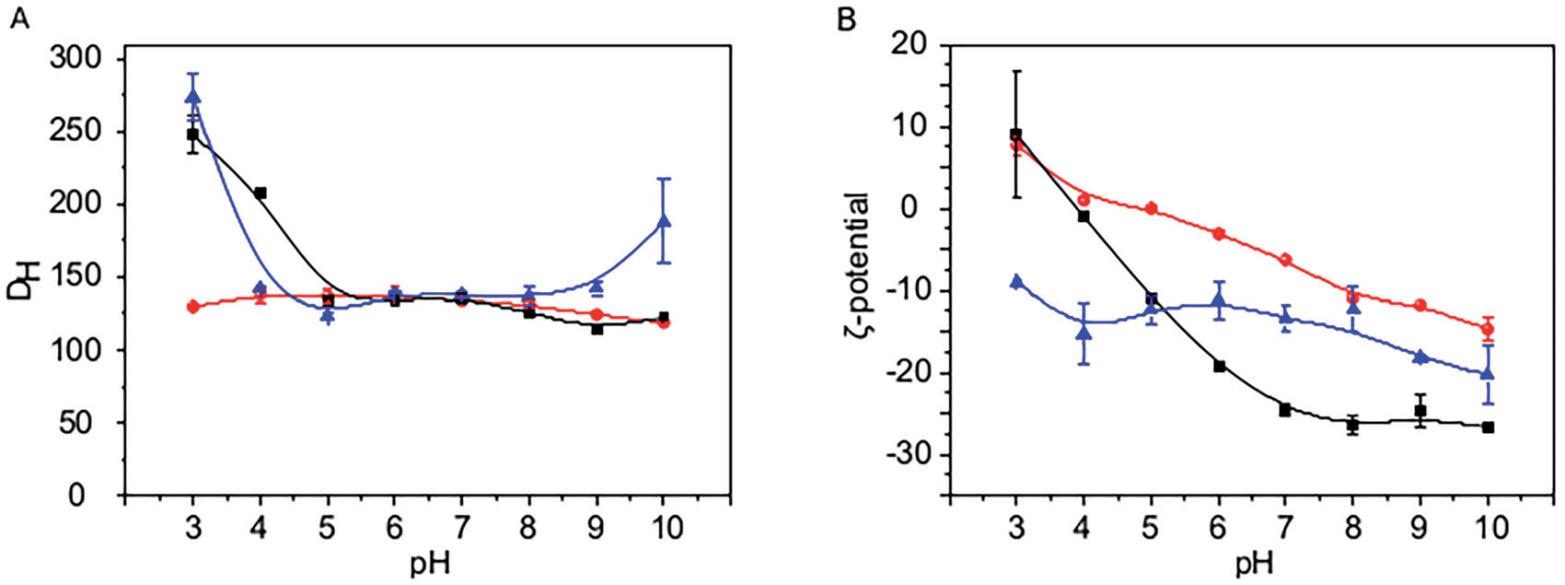
Colloidal characterization of SLNs at different pH values. (A) *D*_H_ and (B) *ζ*-potential of (red line and circles) PMA, (black line and squares) PCMA, and (blue line and triangles) PCMA-HA. SLNs (10 μL) were diluted in 1 mL of the corresponding buffer (Table SM1), incubated for 30 min, and analyzed by DLS. All measurements were carried out in triplicate at 25 °C. The *ζ*-potential was calculated according to the Smoluchowski theory. Data appear as the mean value ± standard deviation.

The three types of MA SLNs, namely PMA, PCMA, and PCMA-HA, were successfully prepared according to Section 2.3. After the cleaning process, final MA concentrations from PMA, PCMA, and PCMA-HA were 7.5 mg/mL with encapsulation efficiencies above 90% in all cases. Figure 1 shows the colloidal characterization of the three SLNs. All of them were stable in a wide range of pH, since the *D*_H_ of PMA remains between 120 and 140 nm along the whole pH range assayed, PCMA shows *D*_H_ between 110 and 140 nm from pH 5 to pH 9, and PCMA-HA shows *D*_H_ between 120 and 140 nm from pH 4 to pH 9. The *D*_H_ of PCMA and PCMA-HA suffer a slight increase at acidic pH values with PCMA exhibiting a *D*_H_ of 248 ± 13 nm at pH 3 and 208 ± 3 nm at pH 4, and PCMA-HA exhibiting a *D*_H_ of 274 ± 16 nm at pH 3. However, the average *D*_H_ always remains below 300 nm, providing evidence of the stability of all three colloidal systems. TEM images from SLNs (Fig. SM1) show the spherical shape of the system and confirms that sizes match *D*_H_ values obtained by DLS. Changes on the *ζ*-potential were noted with variations from a positive value at pH 3 for PMA and PCMA, to negative values as the pH increased. The increase on the negative *ζ*-potential of PCMA was observably higher compared to that of PMA. PCMA-HA exhibited a notably different behavior with negative *ζ*-potential values along the whole pH range.

### 3.2. Colloidal properties of MA SLNs after *in vitro* digestion

**Figure 2.**
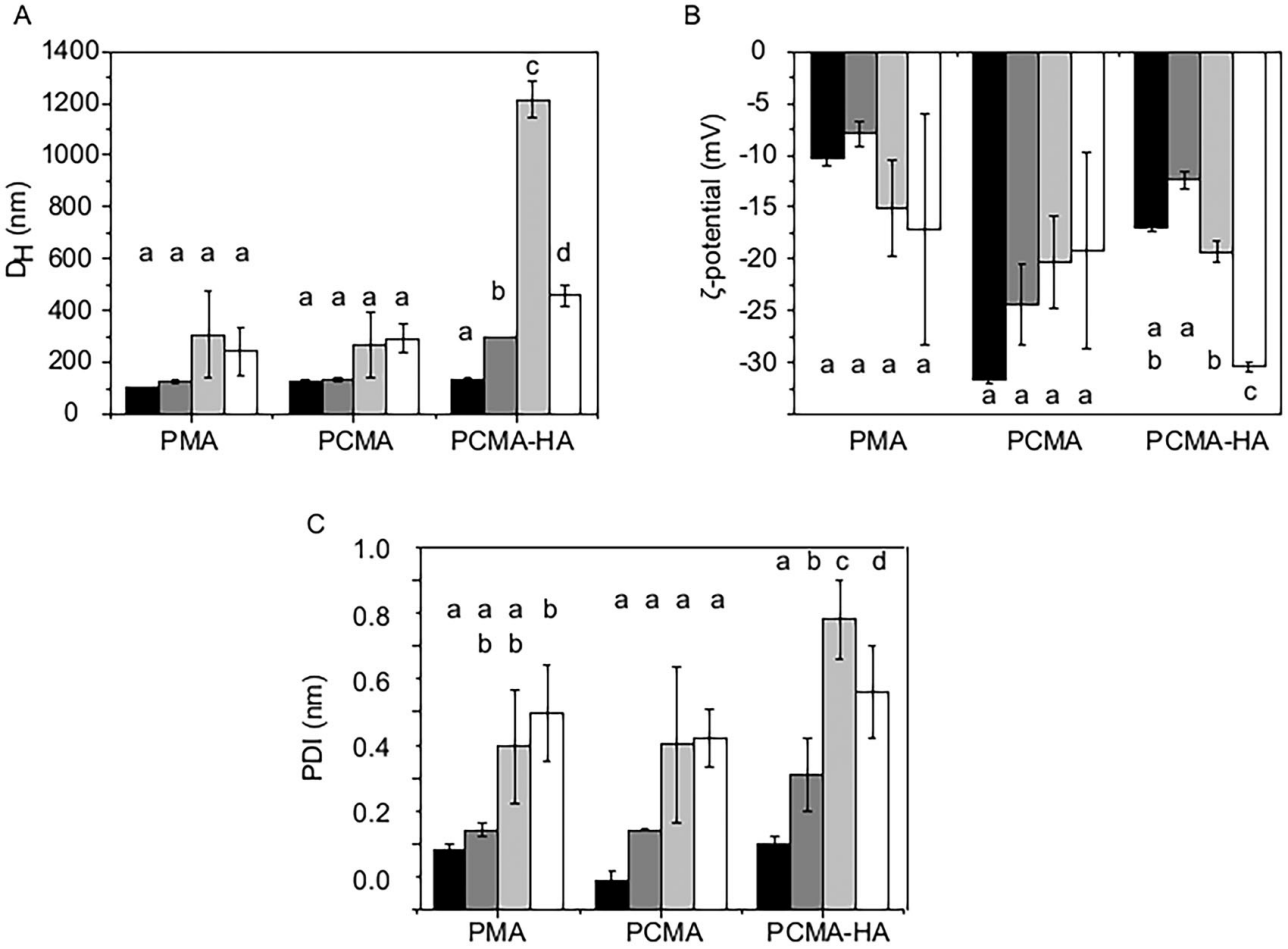
Colloidal characterization of the SLNs before and after the GI digestion process. (A) *D*_H_, (B) *ζ*-potential, and (C) PDI of PMA, PCMA, and PCMA-HA. (Black) Initial state before digestion, (dark grey) oral digesta, (bright grey) gastric digesta and (white) intestinal digesta. Ten microliters of SLNs were diluted in 1 mL of pH 7 buffer (KH_2_PO_4_, 1.13 mM), incubated for 30 min, and analyzed by DLS. All measurements were performed in triplicate at 25 °C and the self-optimization routine from the instrument software was used. The *ζ*-potential was calculated according to the Smoluchowski theory. Data appear as the mean value ± standard deviation. One-way ANOVA statistic test followed by Bonferroni’s multiple comparison post hoc test was applied (*p*<0.05). Significant differences between digestive samples from the same SLNs are indicated with different letters.

To monitor the stability of the three SLNs along the *in vitro* digestion process, we analyzed the colloidal parameters of the nanoparticles after the different digestion phases: oral, gastric, and intestinal (Figure 2). Digestive solutions (bile salts, enzymes, and enzymes inhibitors) were removed by filtration, and the SLNs in the retentates were diluted with a low ionic strength buffer solution (KH_2_PO_4_, 1.13 mM, pH 7) prior to the measurements.

After the oral phase, we observed an increase in *D*_H_ only in the case of PCMA-HA, suggesting that this system is more sensitive to ionic strength than PMA and PCMA. As the digestion continued, we observed a slight increase in *D*_H_ and PDI in PMA and PCMA, but it was statistically significant (*p*<0.05) for PCMA-HA, mainly during the gastric step. The later size reduction in the intestinal phase suggests that PCMA-HA was suffering flocculation at the gastric phase. At the end of the digestion, PMA and PCMA had a similar average *D*_H_ (240 ± 90 nm and 290 ± 50 nm, respectively), which is significantly less (*p*<0.05) than that of PCMA-HA (460 ± 40 nm).

The *ζ*-potential of all SLNs types was also altered during *in vitro* digestion process. The more remarkable change was that of PCMA-HA after the intestinal step (–30.4 ± 0.5 mV, Figure 2(B)). As in the case of *D*_H_, PCMA-HA suffered higher changes on the *ζ*-potential.

MA was quantified by LC–MS/MS before and after the GI digestion. At the end of the intestinal phase, MA as a percentage of initial MA was 55 ± 22% for PMA GI, 55 ± 14% for PCMA GI and 75 ± 24% for PCMA-HA GI. When the same process was performed on free MA, less than 1% was recovered post GI digestion.

### 3.3. Permeability results

Since MA exhibits anti-cancer activity, the toxicity of the SLNs on Caco-2 cells was checked to ensure that the monolayer was not damaged during the permeability experiments. With this aim, undifferentiated Caco-2 cells were treated during 4 h with GI digested PMA and PCMA SLNs at different concentrations. Upon analysis with MTS assay, more than 80% of cell viability was observed even in the case of the highest MA concentration tested, 400 μM (Fig. SM2).

**Figure 3.**
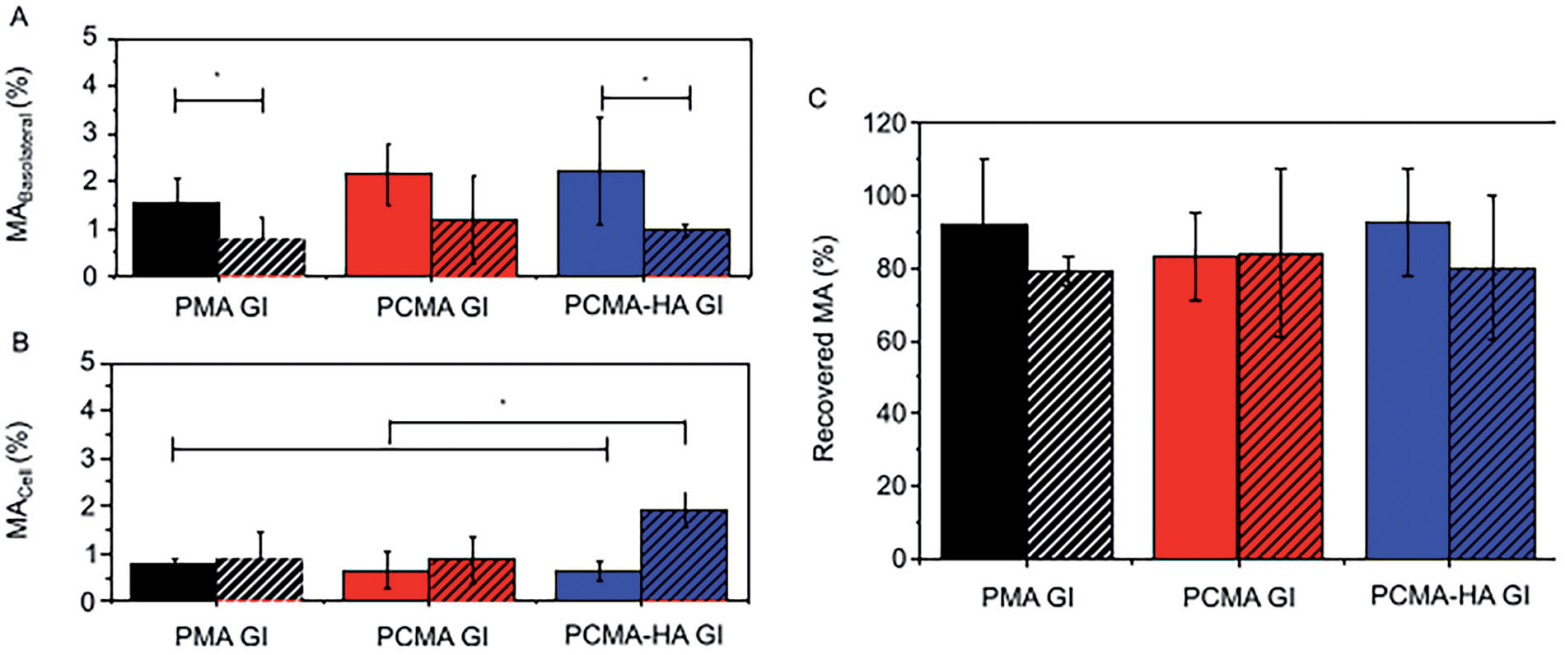
Percentage of MA recovered from (A) the basolateral fraction, (B) cellular fraction, and (C) apical + basolateral + cell fraction samples after 4 h permeability in 21 days old (solid columns) Caco-2 or (striped columns) Caco-2/HT29-MTX (75:25) monolayers treated with PMA GI, PCMA GI and PCMA-HA GI. Final apical volume 0.5 mL and final basolateral volume 1.5 mL. MA was quantified by LC MS/MS. Data are expressed as a % of MA initially added to the apical chamber, and are the average value of at least three independent replicates. Error bars indicate the standard deviation. One-way ANOVA test and Bonferroni’s correction were applied. Significant differences are highlighted with an asterisk ‘*’ (*p*<0.05).

To examine the permeability of MA to cross the gut barrier, GI digested PMA, PCMA and PCMA-HA were applied for 4 h to the apical side of 21 days old polarized Caco-2 monolayers or 21 days old Caco-2/HT29-MTX 75:25 co-cultures secreting a mucus layer. After this differentiation in transwell plates, TEER values ranged from 1,000 to 2,100 Ω·cm^2^ in Caco-2 monolayers and from 550 to 1,500 Ω·cm^2^ in Caco-2/HT29-MTX monolayers, indicating the formation of tight junctions. Initial concentrations of MA applied to the apical chamber were 150 ± 30 μM in PMA, 145 ± 40 μM in PCMA GI and 120 ± 30 μM in PCMA-HA for Caco-2. The subsequent basolateral concentrations of MA were 2.4 ± 1.3 μM with PMA GI, 3.3 ± 1.2 μM with PCMA GI and 2.5 ± 1.1 μM with PCMA-HA-GI. For Caco-2/HT29-MTX models, the MA concentrations applied were 140 ± 30 μM in PMA, 150 ± 40 μM in PCMA GI and 159 ± 9 μM in PCMA-HA. The basolateral MA concentrations were 1.1 ± 0.6 μM with PMA GI, 1.8 ± 1.6 μM with PCMA GI and 1.4 ± 0.2 μM with PCMA-HA-GI. The MA concentrations reaching the basolateral chamber as a percentage of the concentrations applied to the apical are presented in Figure 3(A). In the Caco-2 model, PCMA-HA-GI showed a permeability of 2.2 ± 1.1%. The apparent permeability coefficient (*P*_app_) for PCMA-HA-GI in the Caco-2 model was 6.8 × 10^−7^±3.5 × 10^−7^ cm/s (Table SM2). In the Caco-2/HT29-MTX model, PCMA-HA-GI report a permeability of 1.0 ± 0.1% (*P*_app_=2.9 × 10^−7^±0.4 × 10^−7^ cm/s) (Table SM2). Comparing results from both epithelial models, we observed significant reductions in the permeability of all three MA SLNs in the mucus-producing Caco-2/HT29-MTX co-culture compared to the Caco-2 model (*p*<0.05).

MA concentration was also determined in the cellular fraction as a percentage of the initial apical concentration (Figure 3(B)). Here, we observe that only in the case of PCMA-HA-GI there is a significant (*p*<0.05) difference between both models, with a higher retention in cells in the Caco-2/HT29MTX co-culture (1.9 ± 0.4 μM) compared to Caco-2 model (0.7 ± 0.2 μM, *p*<0.05) and compared to all other samples. For the other two SLNs, there were no significant differences in cellular fraction content of MA in Caco-2 or Caco-2/HT29-MTX cells.

When comparing the initial amount of MA added to the apical chamber to the final amount recovered after the assay (apical + basolateral + cellular) (Figure 3(C)), we observed MA loss which could not be explained by the quantity in the cellular and basolateral fractions.

### 3.4. Pancreatic cancer cells uptake of MA SLNs after crossing the gut barrier

**Figure 4.**
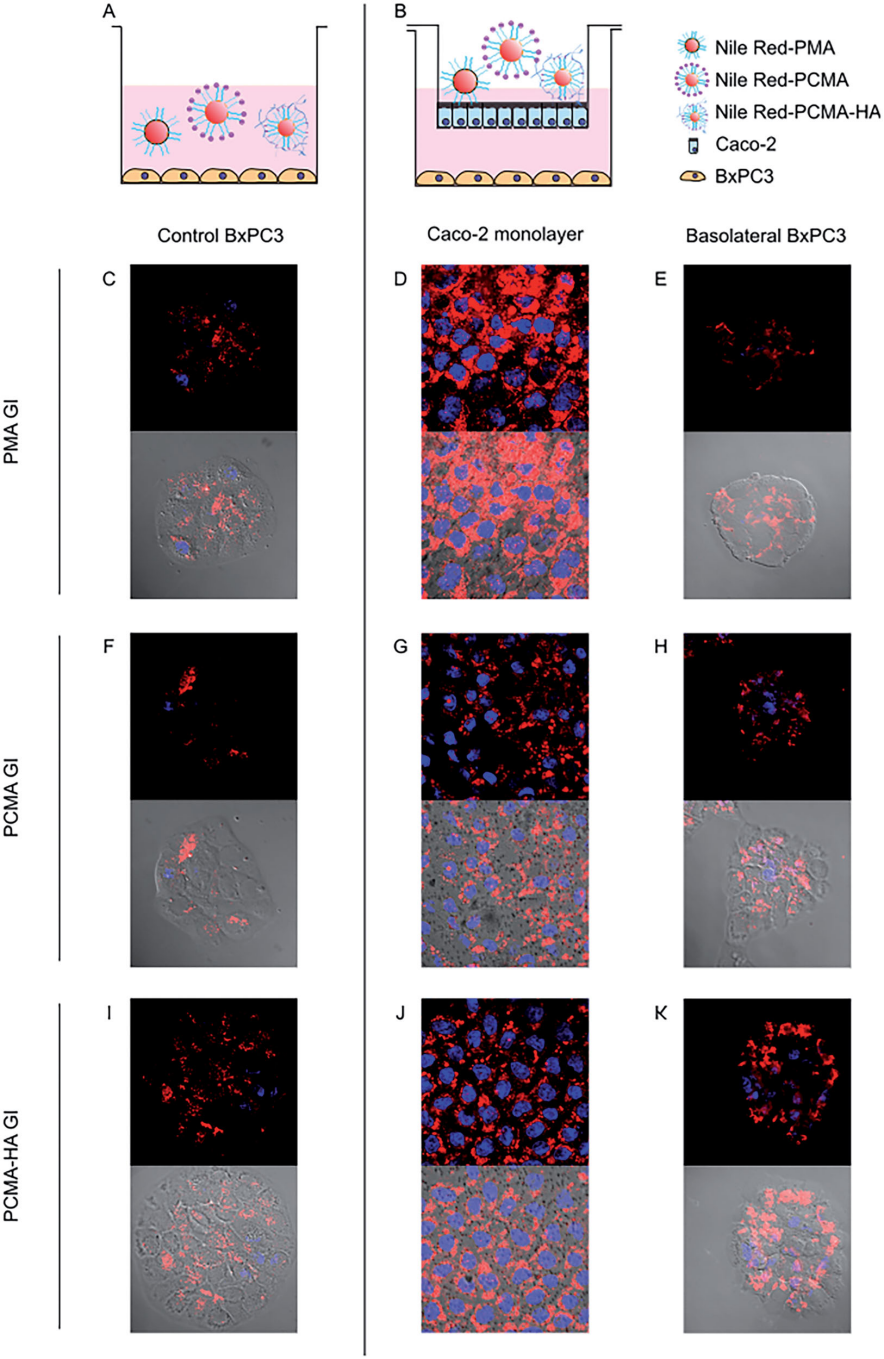
(A, B) Experimental design and confocal fluorescence microscopy images of (C, F, I) control BxPC3 cells, (D, G, J) 21 days old Caco-2 monolayers and (E, H, K) BxPC3 cells in basolateral chamber. (Top image) Red and blue fluorescence merged channel and (bottom image) red fluorescence, blue fluorescence and optic image merged channels. Caco-2 monolayers were treated for 4 h with Nile Red loaded gastrointestinal digested SLNs (PMA GI, PCMA GI and PCMA-HA GI) with pancreatic BxPC3 cells present in the basolateral chamber. Apical chamber consisted of 0.5 mL (final apical volume) DMEM with Nile Red loaded PMA-GI, PCMA-GI or PCMA-HA-GI (0.22 mM of Nile Red final apical concentration). Basolateral chamber consisted of 1.5 mL DMEM with 2.5 × 10^4^ BxPC3 cells/well seeded 24 h earlier. Control experiments were BxPC3 cells incubated directly with 0.5 mL DMEM with Nile Red loaded PMA GI, PCMA GI or PCMA-HA GI (0.22 mM of Nile Red) +1.5 mL of DMEM for 4 h. Excitation and emission wave lengths were 543 nm and 651, and 405 nm and 450 nm, for Nile Red and DAPI respectively.

To further investigate if MA SLNs could cross the gut barrier and target tumoral cells, the lipophilic fluorescent compound Nile Red was included in the preparations of the three MA SLNs. These Nile Red loaded MA SLNs were then GI digested. These SLNs were added for 4 h to the apical side of 21 days old Caco-2 monolayers with confluent BxPC3 pancreatic cancer cells on the basolateral side. Confocal fluorescence microscopy was used for tracking the uptake of Nile red loaded SLNs in both cell types (Figure 4(A,B)). Figure 4(C,F,I) shows control images of BxPC3 cells incubated directly with Nile Red loaded SLNs PMA GI, PCMA GI and PCMA-HA GI. Figure 4(D,G,J) shows polarized Caco-2 cells with stained nuclei in blue and Nile Red fluorescence on the cell cytoplasm in red after 4 h treatment. Figure 4(E,H,K) displays the appearance of Nile Red in the BxPC3 cells cultured in the Caco-2 basolateral chamber. GI digested SLNs were taken up by the Caco-2 cells as evidenced by Nile Red fluorescence in the cytoplasm. Moreover, Nile Red-loaded SLNs crossed Caco-2 monolayers, arrived to the basolateral chamber and were taken up by the BxPC3 pancreatic cancer cells. In fact, Nile Red fluorescence found in the cytoplasm of basolateral BxPC3 was similar to that reported in the cytoplasm of control BxPC3 incubated with Nile Red only.

### 3.5. Toxicity of basolateral concentration of MA

**Figure 5.**
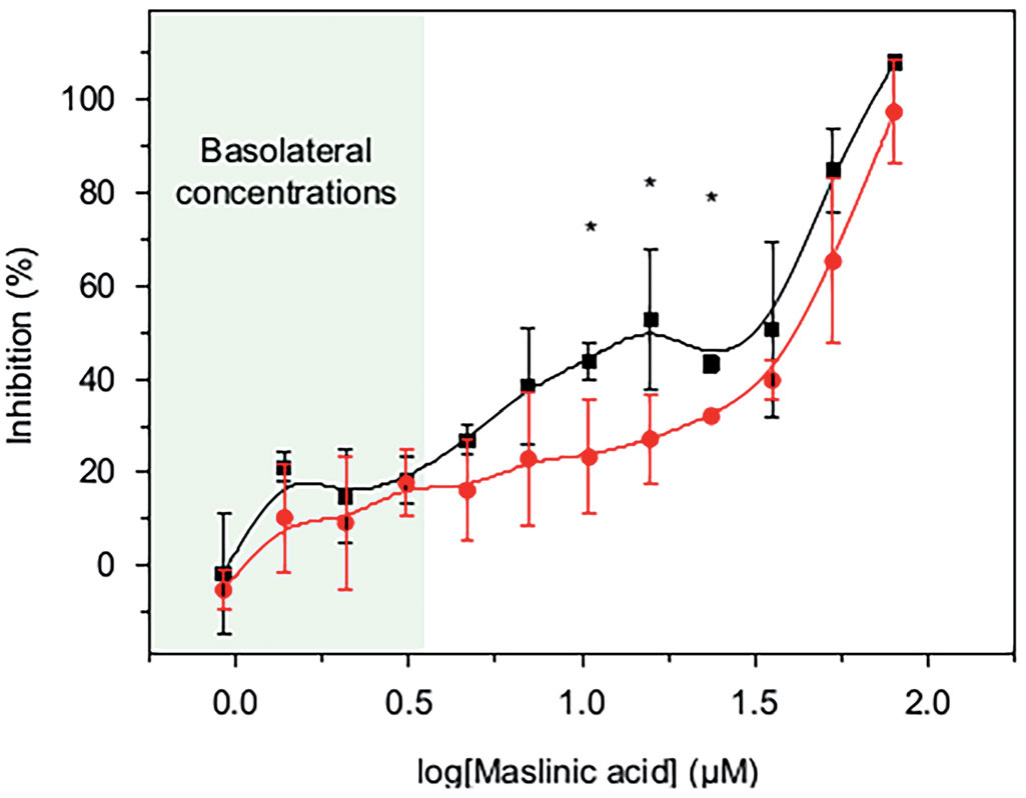
BxPC3 growth inhibition by different concentrations of MA (0.9–79.7 μM) acid over 72 h period. MA was prepared in DMSO and diluted in RPMI. 4.5 × 10^−3^ BxPC3 cells were seeded per well in 96-well plates. After 24 h seeding, the cells were treated with MA test samples. The test samples were refreshed every 12 h (black line and squares) or 24 h (red line and circles). Cell viability at 72 h was measured by MTT. Results are the average value ± standard deviation from three independent assays. Student’s statistic *t*-test (*p*<0.05, two tail and two sample *t*-test for equal variances) was applied for significant differences between the two dosage regimens (12 h or 24 h) for the same MA concentration. Statistically significant differences between regimes (*p*<0.05) are highlighted with ‘*’.

To investigate if MA at the concentrations observed could inhibit the growth of pancreatic tumoral cells, the pancreatic BxPC3 cancer cell line was incubated with varying concentrations of MA (from 0.9 to 79.7 μM) for 72 h (Figure 5). This range was based on the MA concentration quantified in Caco-2 and Caco-2/HT29-MTX basolateral chambers (0.01–3.3 μM, Fig. SM3) and known cytotoxic concentrations of MA. RPMI media were replaced with fresh RPMI with MA every 12 or every 24 h, to simulate a once daily or twice daily drug dosage regime, respectively. The IC_50_ values were regime dependent, with IC_50_^12h^ at 19 ± 3 μM MA and IC_50_^24h^ at 37 ± 3 μM. Differences between the DMSO treated cells used as control and the range of MA concentrations assayed were statistically significant (*p*<0.05) from 1.4 μM on the 12 h regime and 3.1 μM on the 24 h regime. By replenishing with fresh RPMI plus MA every 12 h, the growth inhibition was significantly higher at MA concentrations of 23.6 μM, 15.7 μM and 10.5 μM compared in a 24 h dose regime (*p*<0.05).

The basolateral concentrations of MA (1.4–3.3 μM) significantly reduced the BxPC3 cells viability with respect to the DMSO treated cells used as control (*p*<0.05). The growth inhibition was 21 ± 3% for 1.4 μM MA with twice daily drug dosage regime (12 h), and 18 ± 7% for a concentration of 3.1 μM with the once daily drug dosage regime (24 h).

## 4. Discussion

In this study, we have shown that MA can be successfully formulated in the form of colloidal NPs, in particular SLNs. To be colloidally stable, these MA SLNs must be surrounded by a surfactant shell and the composition of this shell represents the exposed surface of the system, which is, in fact, responsible for the differences in colloidal characteristics and stability between SLNs. The three nanocarriers studied, namely PMA, PCMA and PCMA-HA, are colloidally stable under a wide range of pH conditions. This remarkable colloidal stability, despite the low *ζ*-potential values exhibited, indicates the role of the steric stabilization set by the nonionic surfactant P407 (Olbrich & Müller, 1999). The small surface negative charge shown by PMA at pH 7 (–6.3 ± 0.5 mV) is a consequence of the carboxylic groups from the MA which build up the core of the SLN. At acidic pH, carboxyl groups are protonated and do not provide SLNs with charge. An increase in pH promotes their deprotonation, which leads to a rise in the negative charge of PMA (from 7.7 ± 1.1 mM at pH 3, to −14.7 ± 1.4 mV at pH 10) and even a higher increase, in absolute values, in the case of PCMA (from 8.93 ± 7.71 mV at pH 3 to −26.7 ± 0.68 mV at pH 10). This noteworthy variation reflects the higher concentration of carboxyl groups on the nanoparticles surface of PCMA, provided by the dicarboxylic-P407. The more stable negative *ζ*-potential of PCMA-HA along with the whole pH range (from −9.1 ± 0.4 mM at pH 3 to −20.3 ± 3.4 mV at pH 10) reveals the presence of HA on the nanoparticle surface. HA has a low pI of 2.5. Therefore, on the assayed pH range from 3 to 10, this molecule remains negatively charged (Kong & Park, 2011; Aguilera-Garrido et al., 2019). This agrees with the observed trend of the PCMA-HA *ζ*-potential. The HA from PCMA-HA shell is also probably responsible for the higher colloidal instability of these nanoparticles during the digestion process.

The formulation of MA in the form of SLNs has allowed us to increase its water solubility by a factor of more than a million (from 3.6 ng/mL up to 7.5 mg/mL). Improving the solubility of MA is key to improving its bioaccessibility. Moreover, these SLN formulations have shown better capacity than free MA to withstand the harsh conditions of GI digestion. After an *in vitro* digestion process of the three MA SLNs prepared in this study, we recovered 54 ± 22%, 54 ± 14%, and 75 ± 24% of MA from PMA, PCMA and PCMA-HA respectively, compared to <1% recovered from the digestion of free MA (not protected in the form of NPs). Digestive enzymes do not modify MA (Lozano-Mena et al., 2016), thus free MA lost in the process may be due to aggregation during the digestion.

When the colloidal parameters of the MA SLNs were studied along the *in vitro* digestive process, some changes were observed. PCMA-HA was the most unstable colloidal system along the whole process, and appeared to flocculate reversibly under gastric digestive conditions, since average size decreased again in the intestinal phase. At the end of the intestinal digestion, all SLNs suffered a slight increase in *D*_H_, revealing a certain level of aggregation. This aggregation may even be responsible for the previously described reduction in MA during the digestive process. However, the size ranges from the three SLNs (242 ± 93 nm PMA, 290 ± 54 nm PCMA and 458 ± 42 nm PCMA-HA) were still suitable for intestinal absorption (Powell et al., 2010; Agrawal et al., 2014). The larger size of PCMA-HA (458 ± 42 nm) could reduce its bioavailability given that size, surface chemistry, and shape of the NPs determine their transport across the intestinal epithelial barrier. Thus, a higher particle size usually leads to a reduction in cellular uptake and intestinal permeability compared to smaller particle sizes (Powell et al., 2010; Agrawal et al., 2014; Babadi et al., 2020).

All MA SLNs crossed the gut barrier models to the basolateral chamber. In particular, fluorescence confocal microscopy images provided evidence that Nile Red loaded into PMA, PCMA and PCMA-HA, post GI digestion, crossed Caco-2 monolayers and were subsequently taken up by BxPC3 cells seeded on the basolateral chamber. However, the presence of the mucus layer in the co-culture model reduced permeability of MA in PMA-GI and PCMA-HA-GI. This reduction in MA in the basolateral fractions of Caco-2/HT29-MTX treated with PCMA-HA-GI samples was accompanied by an increase in the retention of MA in the cellular fraction. HA is a mucoadhesive molecule (Fallacara et al., 2018), whereas P407 is known to improve mucodiffusion of nanoparticles (Schattling et al., 2017; Santalices et al., 2018). We hypothesize that the HA coating prolonged the residence time of PCMA-HA-GI in the mucus layer and, consequently, reduced basolateral MA. In fact, an extended retention in the mucus layer may improve overall MA bioavailability of PCMA-HA (Shrestha et al., 2014). These results indicated shell composition of SLNs will influence their interactions with the intestinal epithelium and given that the lipidic MA core of the SLNs is not susceptible to lipase digestion, the interaction between the shell and the mucus and intestinal epithelium will be the main factor defining the absorption of the compound.

When comparing the initial amount of MA added to the apical chamber for the permeability assay to the final total amount recovered after the assay, we observed MA loss. This could be a consequence of the MA recovery protocol (Peragón et al., 2015) or the metabolization of the compound by cells (Lozano-Mena et al., 2016). In a previous study, Sánchez-González et al. reported an MA bioavailability in plasma of 5.13%, 45 min after a single oral gavage administration of MA (50 mg/kg) in an aqueous solution with (2-hydroxypropyl)-β-cyclodextrin 40% and sodium carboxymethylcellulose 0.5% to Sprague-Dawley rats (*n* = 4) (Sánchez-González et al., 2015). The authors proposed that MA was metabolized by intestinal cells contributing to its low plasma bioavailability. In addition, they ascribed the poor intestinal absorption to the poor water solubility of MA although it was administered together with solubilizing excipients to increase its bioaccessibility (Sánchez-González et al., 2015; Lozano-Mena et al., 2016). In our study, experiments with free MA alone showed <1% was bioaccessible after the GI digestion process. In contrast, the SLNs considerably improved MA bioaccessibility. Moreover, confocal microscopy images showed that GI digested PMA, PCMA and PCMA-HA can facilitate the transport of other hydrophobic compounds, i.e. Nile Red, further highlighting their possible use as hydrophobic drug delivery systems.

Despite low concentrations of MA observed in the basolateral compartment of gut barrier models exposed to SLNs, these concentrations were sufficient to significantly inhibit the growth of the BxPC3 pancreatic cancer cells. Our IC_50_ values agree with inhibitory range concentrations reported in the literature, e.g. for HCT116 colon carcinoma cell line (12 h incubation IC_50_=18.48 μM) (Wei et al., 2019) and HT29 (72 h incubation IC_50_=31.8 μM) (Peragón et al., 2015). On the HepG2 hepatocyte carcinoma human cell line, the IC_50_ at 72 h was estimated at 99 μM (Peragón et al., 2015), on Caco-2 the IC_50_ at 72 h was 86 μM (Reyes-Zurita et al., 2016), and on the B16F10 murine melanoma cell line, the 24 h IC_50_ has been calculated at 42 μM. On the other hand, in A10 healthy rat embryonic cells, no toxic effect has been reported for MA concentrations up to 212 μM (Mokhtari et al., 2020). In our study, viability data from Caco-2 cells and TEER values from polarized monolayers indicated that both Caco-2 and HT29-MTX cells were unaffected by MA concentrations up to 400 μM after 4 h incubation with PMA GI and PCMA GI. This can be explained due to the short incubation time of the SLNs with the Caco-2 and Caco-2/HT29-MTX monolayers (4 h), which is shorter than other reported IC_50_ incubation times.

## 5. Conclusions

MA is a notable anti-cancer natural compound with poor water solubility and bioaccessibility. This study provides evidence that MA can be successfully formulated in the form of SLNs with outstanding improvements in both MA solubility (3.6 ng/mL to 7.5 mg/mL) and bioaccessibility (<1% to 50–75%). Moreover, they can transport and deliver other hydrophobic encapsulated compounds. These MA SLNs, covered with P407, dicarboxylic-P407 or dicarboxylic-P407 + HA, display good colloidal stability in a broad range of pH values. When the SLNs were exposed to a simulated complete GI digestion, more than half of the initial MA remained in the sample, indicating protection during gut transit. Post GI digestion, PMA, PCMA and PCMA-HA improved MA transport across *in vitro* gut barrier models. The presence of the mucus layer in the Caco-2/HT29-MTX co-culture model reduced permeability of the SLNs, and the cellular retention of MA was improved with HA covering the nanoparticles (PCMA-HA). Permeability was further proven by confocal microscopy of fluorescent Nile Red loaded MA SLNs. Moreover, the concentrations of MA obtained in the basolateral compartment from *in vitro* gut barrier models were able to inhibit the growth of BxPC3 pancreatic cancer cells up to 20%. These results suggest the encouraging potential of SLNs as oral vehicles of MA and indeed other antitumor hydrophobic drugs. Further work should address the *in vivo* bioavailability of MA after oral administration of these promising SLNs.

## Authors’ contributions

Aixa Aguilera-Garrido contributed to conceptualization, methodology, formal analysis, investigation, writing – original draft, and visualization. Elena Arranz contributed to methodology, writing – review and editing, and supervision. Francisco Galisteo-González involved in conceptualization, methodology, resources, writing – review and editing, project administration, and funding acquisition. María José Gálvez-Ruiz contributed to conceptualization, resources, writing – review and editing, project administration, and funding acquisition. Juan Antonio Marchal involved in conceptualization, resources, writing – review and editing, and funding acquisition. Linda Giblin contributed to resources, writing – review and editing, supervision, project administration, and funding acquisition.

## Supplementary Material

Supplemental Material
