# Supplementary material for: Solid lipid nanoparticles to improve bioaccessibility and permeability of orally administered maslinic acid
Source: Drug Deliv. 2022 Jun 28;29(1):1971–82. doi: 10.1080/10717544.2022.2086937 (PMC9246121; doi:10.1080/10717544.2022.2086937)
Supplement: Supplemental Material [file IDRD_A_2086937_SM7558.docx]

**Supplementary material**

**Tables:**

***Buffers composition***

**Table SM1.** Composition of buffers used to analyze the colloidal characteristic of SLNs by DLS (D_H_, PDI and ζ-potential) at different pH. pH was adjusted with NaOH or HCl. Conductivity of all buffers was adjusted with KNO_3_ 1 M to 300 μS/cm.

| pH 3 | Acetic acid 13.5 mM |
| --- | --- |
| pH 4 | Acetic acid 13.5 mM |
| pH 5 | Acetic acid 3.15 mM |
| pH 6 | NaH_2_PO_4_ 1.79 mM |
| pH 7 | NaH_2_PO_4_ 1.13 mM |
| pH 8 | H_3_BO_3_ 150 mM |
| pH 9 | H_3_BO_3_ 5.14 mM |
| pH 10 | H_3_BO_3_ 2.20 mM |

**Apparent permeability coefficient**

***Table SM2****.* Apparent permeability (P_app_, cm/s) of gastrointestinal digested PMA, PCMA or PCMA-HA in Caco-2 and Caco-2/HT29-MTX polarized monolayers. Monolayers were incubated with test samples in 0.5 mL DMEM for 4 h. The initial apical concentrations of MA were: 150 ± 30 μM in PMA, 145 ± 40 μM in PCMA GI, 120 ± 30 μM in PCMA-HA for Caco-2 models and 140 ± 30 μM in PMA, 150 ± 40 μM in PCMA GI, 159 ± 9 μM in PCMA-HA for Caco-2/HT29-MTX models. MA was quantified by LC-MS/MS in basolateral chamber. P_app_ (cm/s) was calculated as P_app_ = $\frac{1}{S\times Q₀}$×$\frac{dQ(t)}{\mathrm{dt}}$, where S is the transwell membrane surface (cm^2^), Q₀ is the initial apical concentration of MA (μg/mL), dQ(t) is the amount of MA on the basolateral chamber after the 4 h incubation (μg), and dt is the incubation time (s). Data are the average value of at least 3 independent replicates ± standard deviation.

|  | Caco-2 | Caco-2/HT29-MTX |
| --- | --- | --- |
| PMA-GI | 4.8 × 10^-7^± 1.6 × 10^-7^ | 2.5 × 10^-7^ ± 1.3 × 10^-7^ |
| PCMA-GI | 6.7 × 10^-7^ ± 1.9 × 10^-7^ | 3.7 × 10^-7^ ± 2.8 × 10^-7^ |
| PCMA-HA GI | 6.8 × 10^-7^ ± 3.5 × 10^-7^ | 2.9 × 10^-7^ ± 0.4 × 10^-7^ |

**Figures:**

**
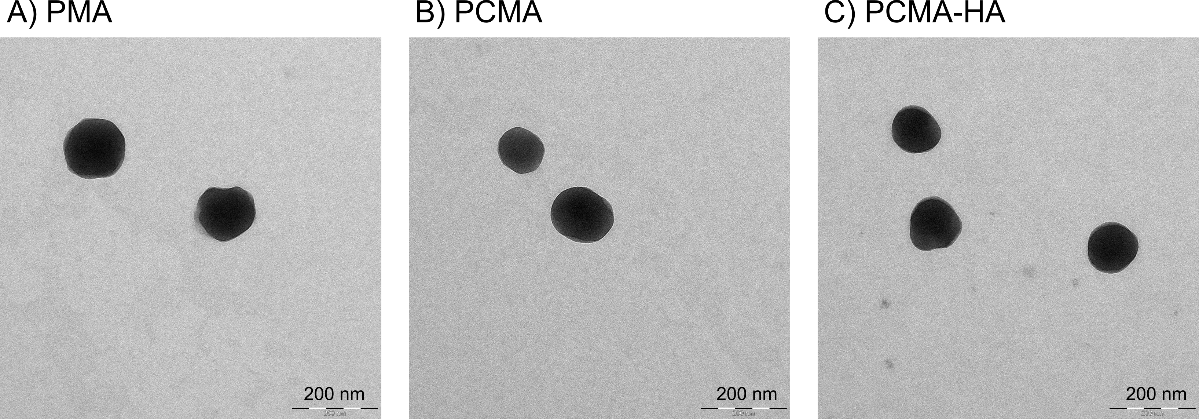
Transmission Electron Microscopy**

**Figure SM1.** High Resolution Transmission Electron Microscopy images of (A) PMA, (B) PCMA, and (C) PCMA-HA. 25 µL of each sample were incubated on carbon-coated grids for 5 minutes before being washed off with ultra-pure water. Uranyl acetate was used as a negative stain. Grids were imaged with HRTEM THERMO FISHER TALOS, 120 kV. SLNs are spherical-shaped, and size and dispersity from images agrees with DLS data (100 – 150 nm).

**
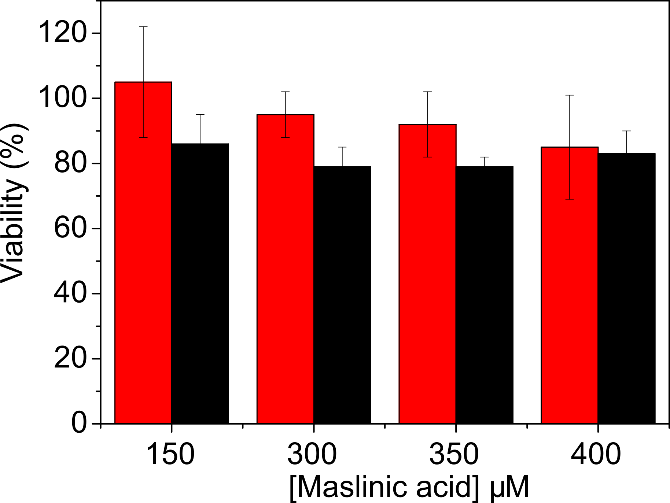
Cytotoxic effect of maslinic acid (MA) in undifferentiated Caco-2 cells after 4 h incubation**

**Figure SM2**. Cellular viability of undifferentiated adherent Caco-2 cells after incubating for 4 h with different concentrations of (red) PMA-GI and (black) PCMA-GI determined with MTS assay. Caco-2 cells were seeded on 96 well/plates (5.000 cells/well) and allowed to grow overnight. After 2 h of incubation with 100 μL of the corresponding sample, 20 μL of MTS were added per well, and cells were incubated for another 2 h. Then, absorbance was recorded at 490 nm.

**
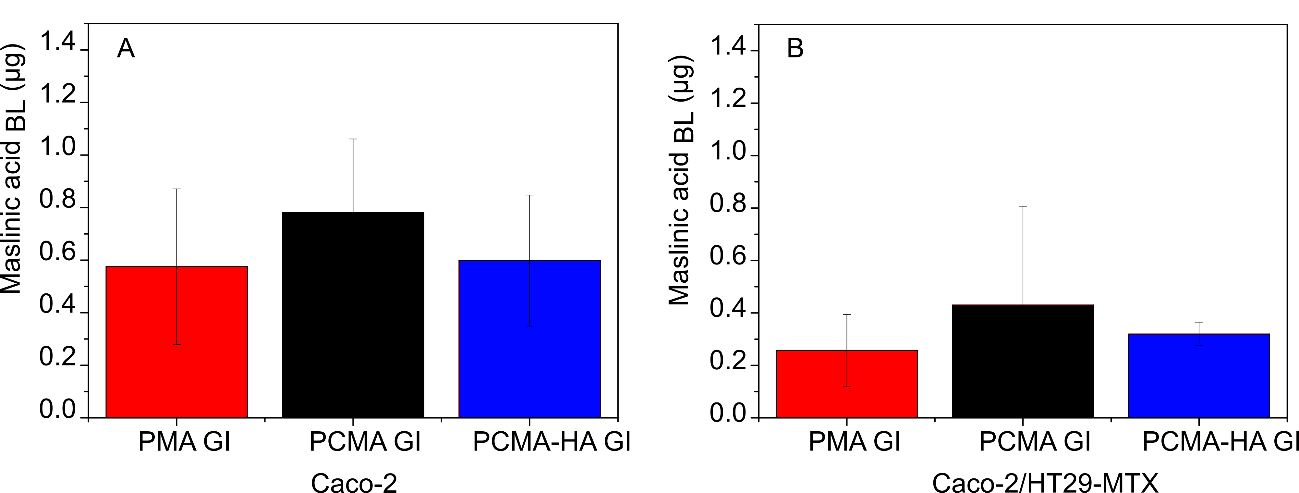
*In vitro* intestinal permeability: Absolute amount of MA**

**Figure SM3**. MA (μg) recovered from the basolateral fraction after the incubation of (A) 21-days old Caco-2 monolayers or (B) Caco-2/HT29MTX (75:25) co-cultures with 0.5 mL DMEM media + (red) PMA GI, (black) PCMA GI or (blue) PCMA-HA GI for 4 h. Basolateral chamber contained 1.5 mL DMEM. MA was quantified by LC-MS/MS. Data are the average value of at least 3 independent replicates and error bars indicate the standard deviation. The statistical analysis was performed with Origin8.


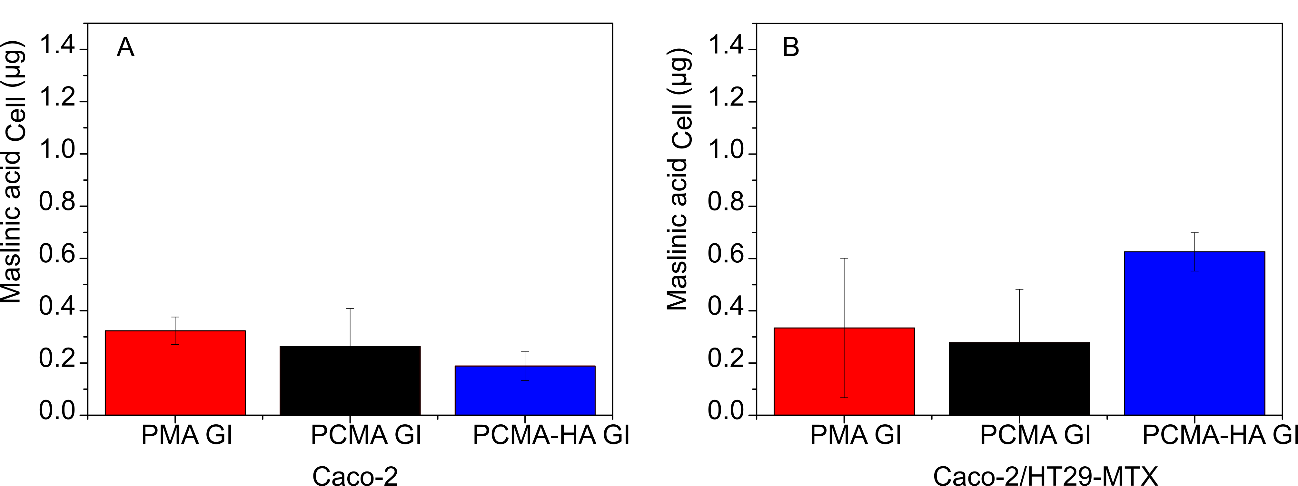


**Figure SM4**. MA (μg) recovered from the cellular fraction after the incubation of (red) PMA GI, (black) PCMA GI or (blue) PCMA-HA GI with the epithelial models for 4 h. (A) Caco-2 epithelial model and (B) Caco-2/HT29-MTX epithelial model were allowed to differentiate for 21 days prior to treatment. TEER was measured before and after the assay to discard samples where the epithelium integrity was compromised. Data are the average value of at least 3 independent replicates and error bars indicate the standard deviation. The statistical analysis was performed with Origin8.


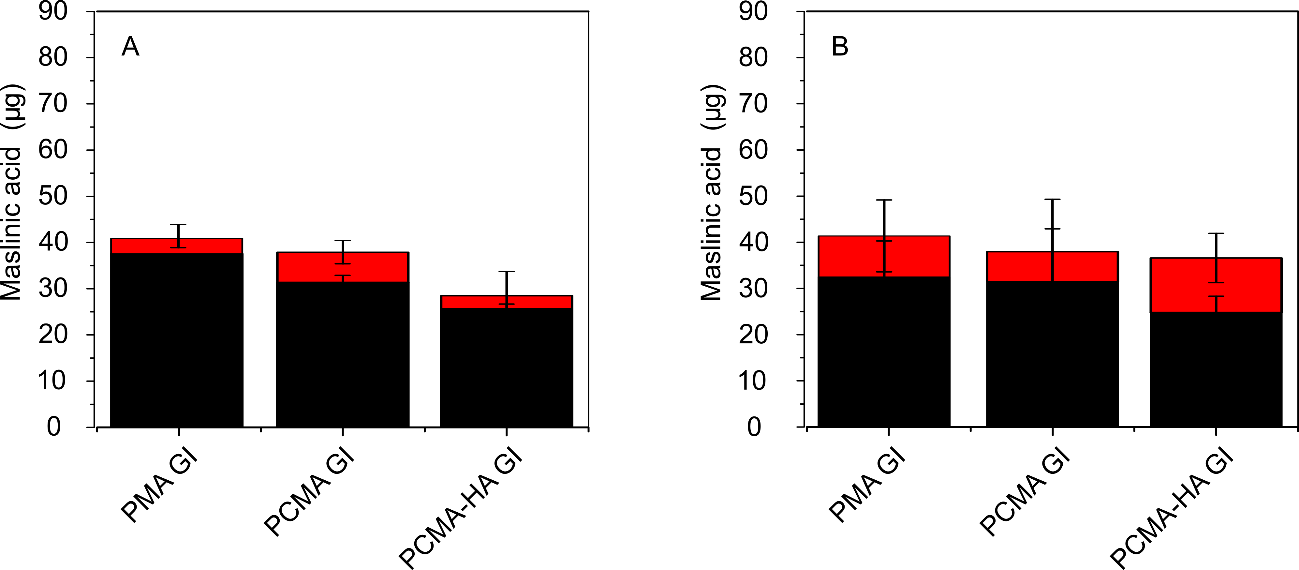


**Figure SM5.** (Red) MA initially added to the apical chamber and (Black) MA recovered after 4 h permeability (apical + basolateral + cell) in 21 days old (A) Caco-2 or (B) Caco-2/HT29-MTX (75:25) monolayers treated with PMA GI, PCMA GI, and PCMA-HA GI for 4 h. MA was quantified by LC-MS/MS. Data are the average value of at least 3 independent replicates and error bars indicate the standard deviation.

**
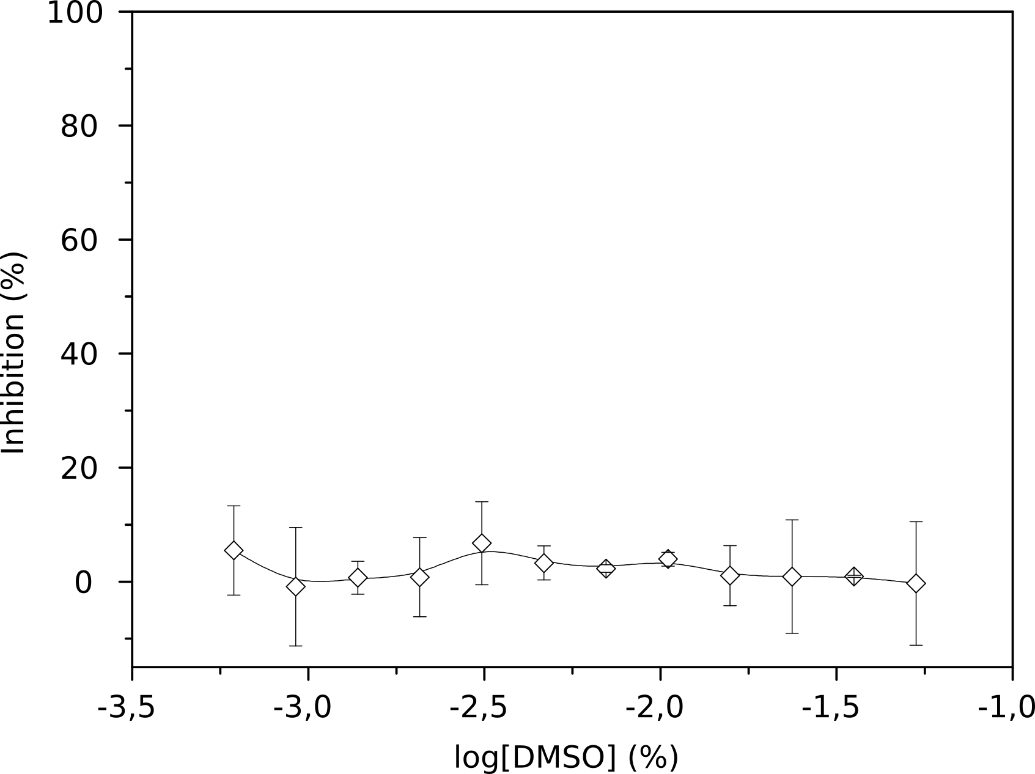
Toxicity of DMSO on BxPC3**

**Figure SM6**. DMSO effect on BxPC3 growth. 4.5×10^3^ BxPC3 cells were seeded per well and allowed to growth for 24 h. Percentages of DMSO match those used to test the toxicity of MA on the cancer cell BxPC3. A stock solution of DMSO 1% in RPMI was prepared. Then, test samples with different percentages of DMSO were prepared and added to BxPC3. The cell viability was evaluated after 72 h of exposition to DMSO with the MTT assay. Results from the figure are the average value ± standard deviation from 3 independent assays.
